# Association of Atypical Antipsychotics With Lipid Abnormalities in Adult Patients With Schizophrenia: A Scoping Review

**DOI:** 10.1002/npr2.70042

**Published:** 2025-09-29

**Authors:** Virginia Abavana, Soban Sadiq

**Affiliations:** ^1^ Kent and Medway Medical School University of Kent Canterbury UK

**Keywords:** atypical antipsychotics, dyslipidaemia, pharmacogenetics, schizophrenia, single nucleotide polymorphism

## Abstract

**Background:**

Atypical antipsychotics (AAs) are commonly used in the treatment of schizophrenia and are often preferred as first‐line therapy over typical antipsychotics (TAs) due to their lower risk of extrapyramidal side effects. Both groups are efficacious in treating symptoms of schizophrenia, but increasing research has highlighted AAs as being associated with a risk of developing dyslipidaemia. Existing research has pointed to the need for more data focusing on the effects of individual AAs on dyslipidaemia in this population.

**Methods:**

The scoping review was conducted using the Preferred Reporting Items for Systematic Reviews and Meta‐Analyses extension for Scoping reviews (PRISMA‐ScR) checklist. The thematic analysis was used to synthesize data from 12 studies selected through structured searches across six databases. Inclusion criteria focused on primary studies between 2015 and 2025 involving adult patients with schizophrenia (18–65 years) treated with AAs and reporting on lipid abnormalities. The themes were identified via Braun and Clarke's six‐step framework.

**Results:**

Clozapine and olanzapine were most strongly associated with increased LDL, total cholesterol, and triglycerides, and reduced HDL. Aripiprazole and lurasidone showed minimal impact. Identified biomarkers included asprosin, IGFBP‐2, MIF, and white blood cell counts. Pharmacogenetic markers such as *APOA1* gene polymorphisms and specific SNPs were also linked to lipid profile variability. Anthropometric indicators like waist‐to‐hip ratio were correlated with dyslipidaemic risk.

**Conclusion:**

The review shows significant associations between specific AAs and lipid abnormalities, particularly with clozapine and olanzapine. Biomarkers and genetic polymorphisms offer promising avenues for monitoring and personalized treatment. Evidence for certain AAs, such as amisulpride, paliperidone, and ziprasidone, remains sparse, highlighting the need for further targeted research. These findings support informed prescribing and the development of predictive tools to mitigate metabolic risks in the treatment of schizophrenia.

## Abstract 2

This scoping review highlights the link between atypical antipsychotics and dyslipidaemia in adults with schizophrenia, identifying clozapine and olanzapine as high‐risk agents. Biomarkers and genetic polymorphisms offer promise for personalized treatment. Further research is needed on lesser‐studied drugs and predictive tools.

## Graphical abstract

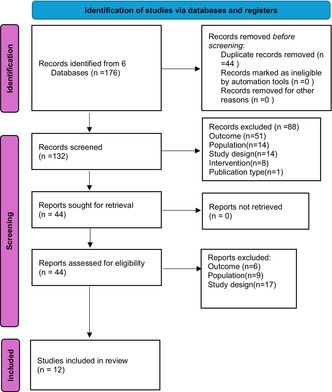

**Abbreviations**
AA: atypical antipsychotics
APOA1: apolipoprotein A1
HDL: high‐density lipoprotein
IGFBP‐2: insulin‐like Growth Factor Binding Protein 2
LDL: low‐density lipoprotein
MIF: macrophage Migration Inhibitory Factor
SNP: single nucleotide polymorphism
TA: typical antipsychotics

## 1 Introduction

Atypical antipsychotics (AAs) are commonly used to treat patients with schizophrenia. AAs have been shown to improve quality of life in this population, decreasing hospital admissions and mortality rates [1]. However, it is estimated that 40% of patients with chronic schizophrenia experience metabolic syndrome, and increasing evidence associates AAs with cardiometabolic adverse effects, including dyslipidaemia [2]. Metabolic syndrome is defined by the World Health Organization as'the presence of insulin resistance (impaired fasting glucose, impaired glucose tolerance, or type 2 diabetes mellitus) in addition to two of the following risk factors: obesity (waist–hip ratio or body mass index), hyperlipidaemia (hypertriglyceridemia, low high‐density lipoprotein [HDL] cholesterol), hypertension, or microalbuminuri' [3, 4]. Regarding lipid levels, it is classified as increased triglycerides > 150 mg/dL and reduced HDL of < 40 mg/dL [3].

Dyslipidaemia is defined as “the imbalance of lipids such as cholesterol, low‐density lipoprotein cholesterol, (LDL), triglycerides, and high‐density lipoprotein (HDL)” [5]. Patients with schizophrenia treated with AAs such as clozapine, olanzapine and quetiapine have been shown to have higher levels of triglycerides and lower HDL levels than those treated with risperidone or aripiprazole [6, 7]. Indeed, cardiovascular disease is a prominent cause of premature morbidity in patients treated with AAs, reducing life expectancy by 10 to 20 years [8]. These consequences highlight the need to further investigate the underlying causes of dyslipidaemia development in adults with a diagnosis of schizophrenia who are treated with AAs. This would help with prescribing choices and could potentially highlight useful biomarkers to predict how particular individuals react to AA use. Additionally, highlighting the effect of individual AAs on developing dyslipidaemia also assists prescribing choices. National prescribing guidelines in the UK do not recommend the use of a particular antipsychotic as a first‐line treatment option for adults with schizophrenia. The *National Institute for Clinical Excellence* (NICE) recommend that patient choice and individual characteristics should guide prescribing choices [9]. This further highlights the importance of gaining a deeper understanding of the association to facilitate appropriate AA prescribing, with the aim to improve patient quality of life and mitigate the cardiometabolic consequences of AA‐induced dyslipidaemia in the population.

### 1.1 Literature Review

AAs are commonly used as a first‐line treatment option in adults with schizophrenia in the UK [10]. Overall, AAs are similarly efficacious to TAs in managing the symptoms of schizophrenia in adults. However, the extrapyramidal adverse effects (EPSE) commonly experienced by patients treated with TAs, such as tardive dyskinesia, can affect compliance with medication [11]. AAs are not commonly associated with such adverse effects; however, they also have specific adverse effects. Numerous bodies of evidence, notably the *Clinical Antipsychotic Trials of Intervention Effectiveness* (CATIE) study, showed a link between AA use and the development of metabolic disorders including dyslipidaemia and obesity in adults with schizophrenia [12]. These increase the patient's risk of cardiovascular disease and mortality [13]. However, as recently as 2024, the underlying mechanisms leading to dyslipidaemia in patients treated with AAs have been described as “inadequately elucidated” [14]. Therefore, there is a huge potential to mitigate AA‐induced dyslipidaemia and its associated consequences on morbidity and mortality if underlying mechanisms are identified. Identifying specific causes could facilitate the development of personalized medicine and identify new therapeutic or prophylactic targets in the population. Possible underlying contributing factors could include cellular disruptions in cholesterol uptake, trafficking, and metabolism by AAs [15]. Additionally, gene polymorphisms affecting the APOA1 protein, a protein involved in cholesterol re‐uptake by the liver [16], have been shown to affect the severity of dyslipidaemia experienced by adult patients with schizophrenia treated with AAs [17]. Additionally, a variation in the extent of lipid dysregulation has been identified between the different agents within the AA group [7].

Existing research shows a clear link between the development of dyslipidaemia and treatment with AAs in adult patients with schizophrenia. However, numerous bodies of evidence have highlighted that the mechanisms underlying AA‐induce dyslipidaemia in humans are poorly understood [14, 18]. Additionally, variations in the extent of dyslipidaemia among patients treated with different AAs within the group have been noted. It has been recommended that more data is needed for individual AAs, as many studies pool data for the entire AA class or for groups of AAs [7]. Scoping reviews are conducted to “map the extent, range, and nature of the literature, as well as to determine possible gaps in the literature on a topic” [19]. Therefore, with regards to the association of AAs with the development of dyslipidaemia in adults with schizophrenia, a scoping review is suited to address the dilemmas highlighted above. Firstly, it allows for the identification of the current literature available for each individual AA used to treat schizophrenia and the extent of dyslipidaemia seen with each. From this, the scoping review highlights which AAs are more/less researched, and which AAs have the most/least significant effect on dyslipidaemia in adults with schizophrenia. Secondly, the nature of a scoping review allows for assessment of the range and nature of literature regarding the current understanding of underlying mechanisms of dyslipidaemia development in adults with schizophrenia following AA use.

### 1.2 Research Question

The research question to be answered by the study is “How are atypical antipsychotics associated with the development of lipid abnormalities in adult patients with schizophrenia?” *The Population, Intervention, Comparison, Outcome and Time* (PICOT) framework was used to generate this research question (Table 1). Using a structured format such as PICOT has been shown to increase the precision of results yielded by researchers [20].

**TABLE 1:** Development of research question with PICOT framework.

PICO	
Population	Adult patients with schizophrenia
Intervention	Atypical antipsychotic medication
Comparison	Patients taking alternative antipsychotic medication, or no medication
Outcome	Lipid abnormalities
Time	Within the last 10 years
Research question	“How are atypical antipsychotics associated with the development of lipid abnormalities in individuals with schizophrenia?”

### 1.3 Aim

The aim of this research was to investigate and establish current understanding surrounding the association of AAs with the development of lipid abnormalities in patients with schizophrenia.

### 1.4 Objectives

1. To determine the association of individual AA on the development of lipid abnormalities in adult patients with schizophrenia.
2. To identify existing understanding of underlying mechanisms involved in the development of lipid abnormalities, to guide future research into these as possible prophylactic or therapeutic targets.
3. To identify gaps in the current knowledge regarding the effect of individual AAs on the development of lipid abnormalities; to identify possible areas for future research.

### 1.5 Search Strategy

The search strategy for the scoping review was based on The Preferred Reporting Items for Systematic reviews and Meta‐Analyses extension for Scoping reviews (PRISMA‐ScR) checklist [21]. The completed checklist can be viewed in a Supporting Information. Following structured guidelines when conducting research enhances methodological transparency and aims to improve the quality of scoping reviews [21].

### 1.6 Eligibility Criteria

The PICO (*Population, Intervention, Comparison, and Outcome*) set for the review (Table 1) was used to guide the parameters for the inclusion criteria. The use of the PICO framework is recommended to facilitate a comprehensive search of literature. These parameters were used to guide the development of the inclusion and exclusion criteria for the study (Table 2). The systematic use of inclusion and exclusion criteria impacts the external validity of the study [22].

**TABLE 2:** Inclusion and exclusion criteria.

Inclusion criteria	Exclusion criteria
Primary studies of any design. Peer‐reviewed papers, published papers, gray literature. Published between (2015 and 2025) to capture recent developments in the field. Study content guided by PICO: adults diagnosed with schizophrenia, treated with atypical antipsychotics, lipid abnormalities. Participant characteristics: human subjects. Diverse age range for adults (to get broader view)—exclude those only focusing on “young” or “older” adults. Oral formulation of atypical antipsychotics.	Studies not published in English language. Animal studies. Studies focusing on other adverse effects of AAs. Studies focusing only on TAs. Long‐acting injectables.

### 1.7 Information Sources

Scoping reviews explore information from a broad range of literature including peer‐reviewed articles and gray literature [23]. A search of the literature was carried out on the following peer‐reviewed databases.

The search for these was facilitated by keywords, Boolean operators, and modifiers. These are outlined in the Supporting Information, alongside justifications for each term.

### 1.8 Selection of Sources of Evidence

When screening papers for inclusion in a scoping review, it is recommended that titles and abstracts are examined; in the absence of an abstract, the full paper must be read to assess suitability for inclusion [19]. Therefore, in this scoping review, this process was followed by the initial search. It has been suggested that tools such as Rayyan enhance the efficiency of the screening process, assisting with organizing papers for inclusion or exclusion from the study [19]. It was used to screen papers for selection in this scoping review.

**FIGURE 1:**
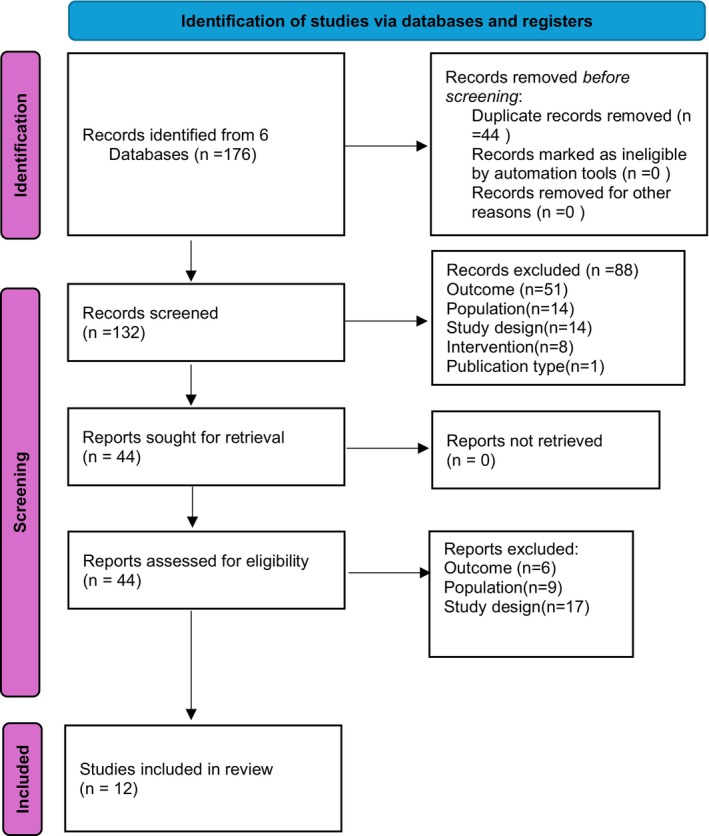
PRISMA flow diagram illustrating the selection process for studies included in the systematic review. A total of Twelve studies met the inclusion criteria and were included in the final review.

As seen in Table 3, an initial search of the databases identified 176 papers. After de‐duplication, 132 papers were chosen for screening by their title and abstract. Following this, 88 papers were excluded, leaving 44 papers for retrieval and full‐text screening. Reasons for exclusion at this stage included the focus of the paper's population, outcome, and study design not aligning with the inclusion/exclusion criteria for the scoping review. After full‐text screening of the 44 papers, 12 were finally included in the scoping review. The final papers selected can be viewed in the Supporting Information. The Preferred Reporting Items for Systematic Reviews and Meta‐Analyses (PRISMA) flow diagram is shown below in Figure 1.

**TABLE 3:** Search databases.

Database	Initial search yield
EMBASE (Ovid)	59
Psych info (Ovid)	12
Cochrane reviews	Reviews = 0; Trial = 29
Scopus Elsevier	39
MEDLINE (PubMed)	37
TRIP database	0
	Total: 176

## 2 Methods

As detailed in the search strategy, a search of the literature spanning six databases was carried out using relevant search terms and Boolean operators. This can be viewed in the Supporting Information. As per the PRISMA flow diagram (Figure 1), 12 papers were finally selected for analysis following de‐duplication, title/abstract screening, and full‐text screening.

### 2.1 Data Charting Process and Data Items

According to the Joanna Briggs Institute (JBI) Manual for Evidence Synthesis [24], it is important to provide a description of the main results of the studies included in a scoping review. Therefore, a charting table with these factors was used to extract data from the studies generated from the search. This can be viewed in a Supporting Information. Data was collected manually by one author and reviewed by another author.

### 2.2 Critical Appraisal

The final selected papers obtained in the search were critically appraised using standard checklists based on the Critical Appraisal Skills Programme (CASP) tools for randomized controlled trials, cohort studies, case–control, and cross‐sectional studies [25], the JBI checklist for case reports [24] and the Centre for Evidence‐Based Medicine (CEBM) checklist for prognostic studies [26], to assess the quality and strength of data obtained. This stage allowed the risk of bias to be assessed. Different appraisal tools were used based on the study design of the papers. Following appraisal, all papers were deemed rigorous enough to be included in the review. The findings of critical appraisal for each paper are summarized in a Supporting Information.

### 2.3 Data Analysis

The specific method used for data analysis in this scoping review was a qualitative, thematic analysis. Thematic analysis is described as a method which “constructs themes to reframe, reinterpret, and/or connect elements of the data” [27]. Indeed, this aligns with the research aims and objectives of the scoping review.

**FIGURE 2:**
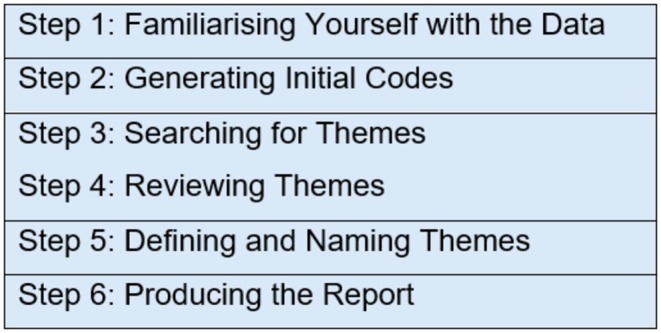
Braun and Clarke's “six steps of thematic analysis.”

To conduct thematic analysis of data, it is recommended to follow six steps as described by Braun and Clarke [28]. These are summarized below in Figure 2.

#### 2.3.1 Step 1: Familiarization With Data

Braun and Clarke [28] recommend starting by familiarizing oneself with the data to enhance a deeper understanding of the data and begin to identify possible patterns. To familiarize oneself with the data, all selected papers were read in their entirety before proceeding to the next stage of data coding.

#### 2.3.2 Step 2: Generating Initial Codes

An initial list of codes was generated by manual annotation and highlighting of the data (Supporting Information). An inductive coding framework was established, in which the data alone was used to identify patterns of note, rather than comparing them to specific existing theories or research. The codes were clearly defined (Supporting Information) and these definitions were applied to all papers included.

#### 2.3.3 Step 3: Searching for Themes

The codes generated in the stage above and their associated data were used to search for wider themes and collated into these accordingly (Supporting Information).

#### 2.3.4 Step 4: Reviewing Themes

As per Braun and Clarke's guide, a two‐step process was used to review the identified overarching themes. Firstly, all coded data associated with each theme was reviewed to confirm relevance and facilitate the identification of any patterns between the data in the theme. Secondly, the whole data set was re‐read to identify or modify the codes/data included under each theme.

#### 2.3.5 Steps 5 and 6: Defining and Naming of Themes and Producing the Report

Following the refinement of codes/data into relevant themes, each theme was defined and described individually, and where relevant associations with other themes were discussed. These descriptions of themes were described in the context of each other (where relevant) and the overarching research question and objectives. The discussion of results also went on to reference relevant literature to place the findings within the existing research available.

## 3 Results

After initial coding of the data in the evidence sources, the following overarching themes were developed (Table 4). A summary of the codes drawn from each paper and how they were used to generate the themes can be viewed in the Supporting Information.

**TABLE 4:** Final themes generated from thematic analysis six‐step process.

Overarching major themes	Minor themes
1. Association of atypical antipsychotics with dyslipidaemia/metabolic effect	Frequency of atypical antipsychotics across included studies
2. Consequences of AA‐induced dyslipidaemia	Anthropometry
3. Potential biomarkers	
4. Pharmacogenetics	

A brief description of each overarching major theme will be given below, followed by the results of the thematic analysis for both major and minor themes (Tables 5, 6, 7, 8, 9, 10).

**TABLE 5:** Table showing main findings of the association of individual AAs with dyslipidaemia.

Paper (first author, date) (reference) and individual AAs included	Effect on individual AAs on dyslipidaemia
Paper 1 (Vázquez‐Bourgon, 2022) [29] Aripiprazole Risperidone	Both AAs cause similar effect on lipid profile over time. Total cholesterol: Both drugs showed a small increase in total cholesterol, but this change was not statistically significant between aripiprazole and risperidone. LDL: Risperidone was associated with a slightly higher increase in LDL cholesterol compared to aripiprazole, though the difference was not large or clinically significant. HDL: There was a small reduction in HDL cholesterol in both groups, but this was not significantly different between the two drugs. Triglycerides: Both aripiprazole and risperidone were linked to small increases in triglyceride levels, with no significant differences between them.
Paper 2 (Li, 2018) [30] Olanzapine Risperidone	Both groups showed a significant increase in triglycerides. LDL increased in both groups, but the increase was higher with olanzapine than risperidone.
Paper 3 (Wysokinski, 2015) [31] Clozapine	Severe, reversible hypertriglyceridaemia.
Paper 4 (Wong, 2024) [32] Clozapine Olanzapine Aripiprazole Risperidone Amisulpride Quetiapine Paliperidone	Specific lipid profile data not published.
Paper 5 (Althanoon, 2021) [33] Olanzapine Aripiprazole	Olanzapine: Induced significant increases in triglycerides, LDL and total cholesterol levels, alongside a decrease in HDL. Aripiprazole: Led to minor, statistically insignificant increases in triglycerides and LDL, with no significant effect on HDL.
Paper 6 (Kang, 2015) [6] Clozapine Olanzapine Quetiapine Risperidone Aripiprazole Ziprasidone	Clozapine: increases in total cholesterol, LDL cholesterol, and triglycerides, even in the absence of substantial weight gain. Olanzapine: there was a strong association with increased triglyceride levels and a decrease in HDL. It also led to increases in total cholesterol and LDL cholesterol. Risperidone: linked to an increase in triglycerides and a modest decrease in HDL cholesterol. Effect less pronounced compared to olanzapine. Quetiapine: showed moderate effects on lipid metabolism, with some increase in triglycerides and total cholesterol, but its effects were less severe compared to olanzapine. Aripiprazole: did not appear to have a significant impact on lipid profiles. It had a more favorable metabolic profile, with minimal effects on cholesterol and triglycerides. Ziprasidone: milder effect on lipid profiles, with only slight increases in total cholesterol and triglycerides, and had minimal impact on LDL and HDL.
Paper 7 (Amini, 2024) [34] Olanzapine Risperidone Aripiprazole	Olanzapine: increased levels of total cholesterol, triglycerides, and LDL while HDL was decreased. Risperidone: increased triglyceride levels and a decrease in HDL cholesterol. However, its effects on total cholesterol and LDL were less pronounced compared to olanzapine. Aripiprazole: minimal impact on triglycerides, total cholesterol, and LDL, and did not significantly alter HDL levels.
Paper 8 (Nolin, 2020) [35] Olanzapine Risperidone	Olanzapine: Significant increase in total cholesterol. Marked increase in triglyceride levels. Increase in LDL cholesterol. Decrease in HDL cholesterol. Risperidone: Significant increase in total cholesterol, though not as pronounced as with olanzapine. Triglycerides: moderate increase. Increase in LDL cholesterol, though the effect was less significant than with olanzapine. HDL: reduced in patients treated with risperidone. Olanzapine‐treated patients showed a higher cholesterol/HDL ratio, and higher levels of triglycerides compared to patients treated with risperidone.
Paper 9 (Correl, 2016) [36] Lurasidone	Patients treated with Lurasidone showed no significant changes in total cholesterol, LDL, HDL, or triglycerides over the long term (22‐ months).
Paper 10 (Chen, 2024) [14] Olanzapine Risperidone Quetiapine Aripiprazole	Olanzapine: Significantly increased total cholesterol levels. LDL cholesterol levels were elevated. Elevated triglycerides. Decrease in HDL cholesterol. Risperidone: Increase in total cholesterol, though the effect was less pronounced than with olanzapine. LDL levels were elevated, but to a lesser degree than olanzapine. HDL cholesterol levels were decreased with risperidone, but the decrease was less than olanzapine. Triglyceride levels were moderately increased. Quetiapine: Moderate increase in total cholesterol. LDL cholesterol was increased, but the effect was less pronounced compared to olanzapine. HDL cholesterol was decreased, but again, the impact was not as severe as with olanzapine or risperidone. Triglycerides showed a mild increase in patients taking quetiapine. Aripiprazole Aripiprazole had minimal effects on total cholesterol. LDL cholesterol showed small changes. HDL cholesterol levels were relatively stable, with no significant decrease. Triglycerides remained stable.
Paper 11 (Fan, 2021) [17] Olanzapine Risperidone Quetiapine Aripiprazole	Olanzapine: Significant increase in total cholesterol levels. LDL cholesterol levels were notably increased in patients treated with olanzapine. Decrease in HDL cholesterol, which is a negative effect on lipid metabolism. Triglyceride levels were also elevated Risperidone: Increase in total cholesterol. LDL cholesterol was elevated, though to a lesser extent than with olanzapine. Decrease in HDL cholesterol. Triglyceride levels were moderately increased with risperidone. Quetiapine: Moderate increase in total cholesterol levels. LDL was slightly elevated in patients on quetiapine. HDL levels were generally decreased with quetiapine treatment. Triglyceride levels were slightly increased with quetiapine. Aripiprazole: Minimal effects on total cholesterol. Little/no change on LDL cholesterol. HDL cholesterol remained stable with aripiprazole treatment. Triglyceride levels remained unchanged with aripiprazole.
Paper 12 (Xiong, 2019) [37] Olanzapine Clozapine	Olanzapine: Significantly elevated total cholesterol levels, which can contribute to increased cardiovascular risk. Increased LDL cholesterol. HDL cholesterol was significantly reduced in patients taking olanzapine. Triglycerides were also significantly elevated with olanzapine treatment. Clozapine: Increase in total cholesterol levels, but to a lesser extent compared to olanzapine. LDL cholesterol levels were elevated with clozapine but less dramatically than with olanzapine. HDL cholesterol was decreased, although the reduction was less severe than olanzapine. Triglyceride levels were moderately increased with clozapine, but the increase was less marked compared to olanzapine.

### 3.1 Association of Atypical Antipsychotics With Dyslipidaemia/Metabolic Effect

This theme explored the well‐known relationship between AAs and the effect on lipid and glucose regulation [2]. The vast majority of evidence utilized to explore this theme focused on data for individual AAs. This was important as it facilitated the identification of AAs more commonly associated with dyslipidaemia and the degree to which they were associated.

### 3.2 Consequences of AA‐Induced Dyslipidaemia

This theme focused on the long‐term consequences of dyslipidaemia induced by AAS, emphasizing the cardiometabolic risks these medications pose to adults with schizophrenia. The results can be viewed below in Table 6.

**TABLE 6:** Table showing main findings of consequences of AA‐induced dyslipidaemia.

Paper (Author, date)	Consequence
Wysokinski (2015)	Severe clozapine‐induced triglyceridaemia
Nolin (2020)	Atherogenesis

### 3.3 Potential Biomarkers/Therapeutic Targets

Various potential biomarkers were identified in the analysis. These are asprosin, Macrophage Migration Inhibitory Factor (MIF), Insulin‐like growth factor‐binding protein 2 (IGFBP‐2) and full blood count (FBC). Biomarkers could be useful to monitor or predict AA‐induced dyslipidaemia in adults with schizophrenia. The results of the findings for this theme can be seen below in Table 7.

**TABLE 7:** Table showing the association of potential biomarkers with dyslipidaemia.

Biomarker (first author, date)	Association with dyslipidaemia
Asprosin (Amini, 2024)	Compared to healthy controls, asprosin levels were elevated in patients treated with AAs. These patients also exhibited dyslipidaemia and insulin resistance.
IGFBP‐2 (Nolin, 2020)	Increased levels of IGFBP‐2 in patients treated with AAs. Negative, significant correlation of circulating IGFBP‐2 levels with BMI, waist circumference and triglyceridaemia. Triglyceridaemia only negatively associated for olanzapine users but not risperidone.
Full blood count (Xiong, 2019)	Elevated white blood cell count (WBC) linked to increased triglyceride levels. Higher platelet counts associated with higher LDL and lower HDL cholesterol. Red blood cell (RBC) count not associated with dyslipidaemia.
MIF (Chen, 2024)	Patients treated with olanzapine, risperidone and quetiapine had higher MIF levels, with associated derangement in lipid profiles. Aripiprazole was associated with lower MIF levels compared to the other atypical antipsychotics, and its metabolic effects were less pronounced.

### 3.4 Pharmacogenetics

This theme focuses on the role of genetic factors in mediating the metabolic side effects of AAs, specifically how individual genetic variations might influence patients' susceptibility to dyslipidemia. By identifying genetic markers associated with lipid changes, pharmacogenetics has the potential to help clinicians personalize treatment. The results can be seen in Tables 8 and 9.

**TABLE 8:** Table showing the association of potential genetic markers with dyslipidaemia.

Gene polymorphism (first author, date)	Effect on dyslipidaemia
*APOA1* −75G/A (Fan, 2021)	Higher triglyceride levels and lower HDL levels in patients on atypical antipsychotics.
*APOA1* 83C/T (Fan, 2021)	Higher total cholesterol and LDL cholesterol levels in patients treated with antipsychotics.
*ABCG2, APOA5, ZPR1, GCNT4, MAST2, and CRTAC1* (Wong, 2024)	Significant link to metabolic changes.

**TABLE 9:** Table showing the association of specific SNPs with dyslipidaemia.

Single nucleotide polymorphism (SNP) (Wong, 2024)	Effect on dyslipidaemia
SNP rs6532055 in the ABCG2 gene	Olanzapine‐induced LDL changes
SNP rs2644520, located near the SORCS1 gene	Aripiprazole‐induced triglyceride changes
SNP rs115843863, near the UPP2 gene	Clozapine‐induced HDL changes
SNP rs2514895, near the KIRREL3 gene	Paliperidone‐induced LDL changes
SNP rs188405603 in the SLC2A9 gene	Quetiapine‐induced triglyceride changes

### 3.5 Frequency of AAs Across Included Studies

**FIGURE 3:**
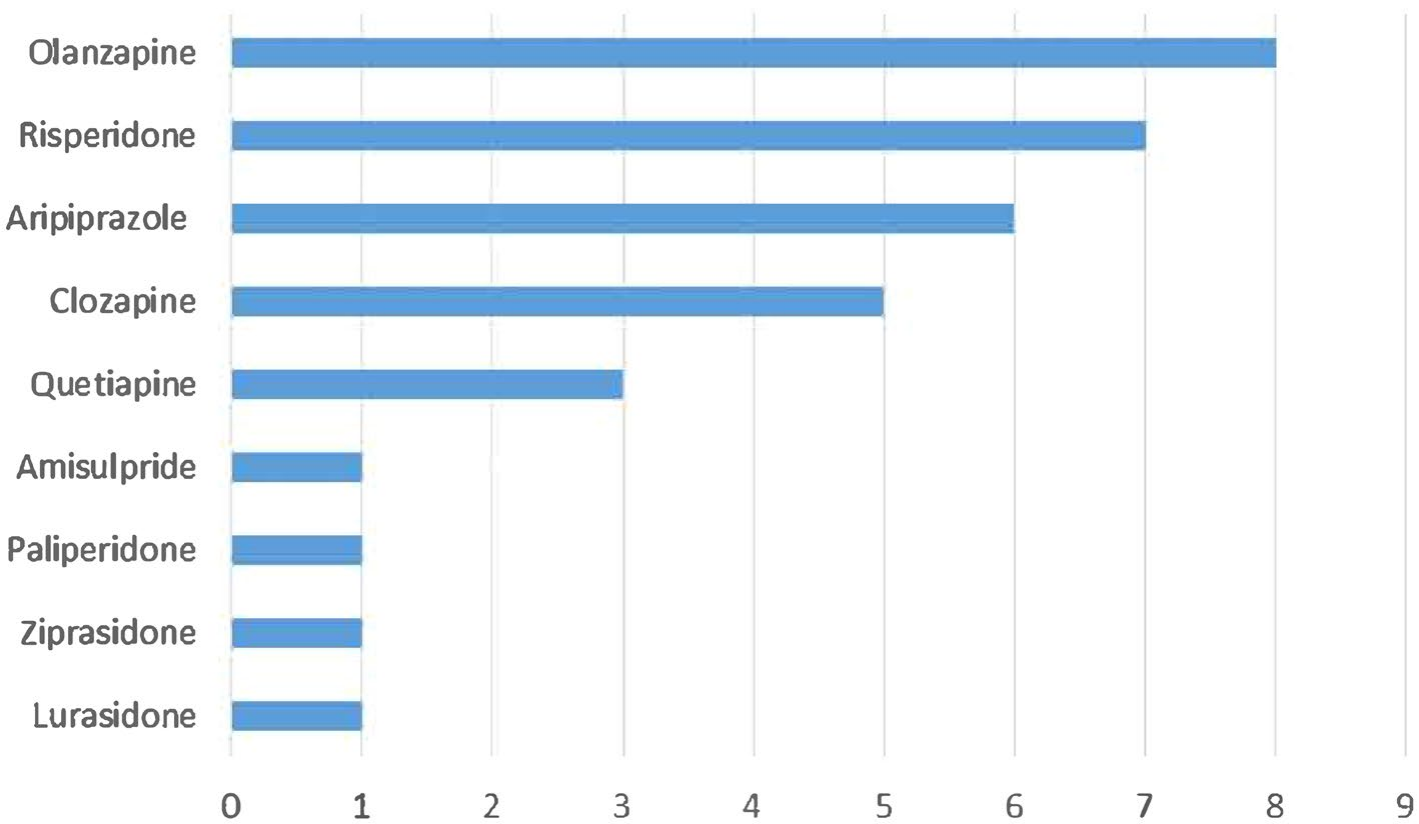
Chart showing the frequency of individual AAs included in the papers selected for scoping review.

Previous research has recommended the need for more research into individual AAs [7]. Therefore, this review has analyzed the spread of data for AAs analyzed in the papers. The data can be seen below in Figure 3.

As shown above in Figure 3, olanzapine, risperidone, and aripiprazole were researched in over half of the papers included in the review. Clozapine was analyzed in 5 of the 12 papers, quetiapine in 3, and lurasidone, asprosin, ziprasidone, paliperidone, and amisulpride were analyzed in one paper.

### 3.6 Anthropometry

The anthropometric features associated with AA‐induced dyslipidaemia are shown in Table 10.

**TABLE 10:** Association of AA‐induced anthropometric changes.

Paper (first author, date)	Anthropometric effect
Li (2018)	Waist: hip ratio was negatively correlated to antipsychotic induced weight gain.
Wysokinski (2015)	Following cessation of clozapine: Reduction in body weight and waist circumference. Reduction in waist‐to‐hip ratio.

## 4 Discussion

### 4.1 Effect of Individual AAs on Dyslipidaemia

This scoping review found that olanzapine and clozapine were most frequently associated with more significant effects on lipid profile derangement compared to other AAs included in the studies. Specifically, they were associated with increases in total cholesterol, LDL, and triglycerides, alongside decreases in HDL. These derangements in lipid profile are one of the criteria for metabolic syndrome, which is known to be associated with adverse consequences such as cardiovascular disease and earlier morbidity [3, 8].

Aripiprazole, lurasidone, and ziprasidone were found to have mild or no significant effect on the development of dyslipidaemia. However, it is important to weigh up the risks alongside the benefits when prescribing AAs. Current guidelines in the UK suggest using any oral antipsychotic medication as a first‐line treatment for adults with schizophrenia [9]. This is with the exception of clozapine, for which there is a strong‐evidence base recommending its use in treatment‐resistant schizophrenia, or where at least two other antipsychotic medications have been trialed and have failed to manage symptoms [10]. Overall, it is recommended to choose an antipsychotic based on individual factors, such as personal choice, risk of adverse effects, and the degree of negative symptoms, with AAs being preferred for negative symptoms [9, 10].

### 4.2 Potential Biomarkers/Therapeutic Targets

#### 4.2.1 Asprosin and Lipid Abnormalities

In the study by Amini et al. [34], asprosin levels were found to be elevated in patients treated with AAs when compared to healthy controls. These elevated asprosin levels were also found to be associated with dyslipidaemia, with patients exhibiting an increase in triglyceride levels and a decrease in HDL levels [34]. Asprosin is a fasting‐induced peptide associated with insulin resistance and metabolic dysfunction. Elevated asprosin levels are strongly linked to obesity, cardiovascular disease, and diabetes [38]. These are hallmark features of the metabolic syndrome often observed in patients receiving AAs [8]. These findings suggest that asprosin may play a role in mediating lipid abnormalities in patients with schizophrenia treated with AAs.

#### 4.2.2 IGFBP‐2 and Lipid Metabolism

Insulin‐like growth factor‐binding protein 2 (IGFBP‐2) was highlighted as another marker associated with lipid metabolism in patients with schizophrenia treated with AAs. IGFBP‐2 is a multi‐functional protein involved in cell signaling and modulation; however, itsexact role in regulating metabolic function is unclear [39]. In the study by Nolin et al. [35] a negative correlation was found between circulating IGFBP‐2 levels and body mass index (BMI), waist circumference, and triglyceridaemia. Patients with IGFBP‐2 below 220 ng/mL showed a comparatively lower incidence of increased waist circumference and lower incidence of elevated triglycerides compared to those above the threshold. This suggests that lower IGFBP‐2 levels may be indicative of a detrimental metabolic response to AAs. This is in line with existing research, which has highlighted IGFBP‐2 as having a protective role in the development of obesity and metabolic dysfunction [39]. It is important to note that the correlation between IGFBP‐2 and triglyceride levels was particularly pronounced for olanzapine users, but not risperidone users [35]. This highlights that the metabolic effects of AAs vary across different medications in the group and emphasizes the need to consider the drug‐specific effects of individual AAs when prescribing AAs.

#### 4.2.3 MIF and Lipid Derangements

Macrophage migration inhibitory factor (MIF) is a cytokine involved in inflammatory responses and has been linked to metabolic dysfunction in various conditions, including obesity and diabetes. In the study by Chen et al. [14] higher MIF levels were observed in patients treated with olanzapine, risperidone, and quetiapine, all of which were associated with deranged lipid profiles. Aripiprazole was associated with lower MIF levels compared to other AAs, indicating that the degree of metabolic disturbance may be linked to the inflammatory burden imposed by these medications. Existing research has identified aripiprazole as having a less significant impact on lipid profile compared to other AAs [6, 39]. The lower MIF levels identified in this study support the existing research and provide a possible pathway via which AA‐induced dyslipidaemia occurs.

#### 4.2.4 Blood Count Parameters and Lipid Abnormalities

Elevated WBC counts were significantly linked to increased triglyceride levels [37]. This could reflect an underlying inflammatory process contributing to lipid abnormalities in patients with schizophrenia treated with AAs. Indeed, existing research has identified inflammatory markers as having a profound role in mediating markers of metabolic dysfunction [40].

Additionally, higher platelet counts were associated with higher LDL cholesterol and lower HDL cholesterol levels [37], further supporting the role of inflammation in lipid derangements. Platelet activation is known to contribute to vascular dysfunction, and its association with unfavorable lipid profiles may further increase the cardiovascular risk in these patients [40]. This information is useful for clinical practice, as the full blood count could highlight patients at higher risk of developing AA‐induced dyslipidaemia.

### 4.3 Pharmacogenetics

Polymorphisms of the *APOA1* gene were identified with a varied prevalence/incidence of the development of dyslipidaemia following the initiation of AA treatment. The *APOA1* gene codes for APOA1, a key protein component of high‐density lipoprotein (HDL) cholesterol, which is involved in reverse cholesterol transport, in which excess cholesterol is removed from tissues and transported to the liver [41]. Two significant gene polymorphisms of *APOA1*, 75G/A and 83C/T, were identified as having association in the development of dyslipidaemia in patients treated with AAs [17]. The *APOA1* 75G/A was associated with higher triglyceride levels and lower HDL levels in patients with schizophrenia treated with AAs. Low HDL cholesterol is a key risk factor for developing cardiovascular disease [3]. The *APOA1* 83C/T polymorphism was associated with higher total cholesterol and LDL cholesterol levels in patients treated with antipsychotics [17]. The association of this polymorphism with elevated LDL cholesterol suggests that individuals with the 83C/T genotype may be more susceptible to the lipid‐altering effects of AAs, especially in relation to drugs that induce substantial lipid changes.

Single‐nucleotide polymorphisms (SNPs) in specific genes have also been linked with the development of lipid abnormalities in patients with schizophrenia treated with AAs (Table 7). These genetic variants are thought to affect drug metabolism, lipid transport, and other metabolic processes, contributing to the variation in lipid responses among different individuals [32].

### 4.4 Frequency of Atypical Antipsychotics Across Included Studies

The studies analyzed in this scoping review looked at the effect of individual AAs on lipid profile, rather than pooling the data for the entire class (Figure 3). This allowed the effects of specific AAs to be compared, highlighting clozapine and olanzapine as having a considerably more detrimental effect on lipid profile compared to other AAs such as aripiprazole, lurasidone, and risperidone.

### 4.5 Anthropometry

The waist‐to‐hip ratio (WHR) is a widely recognized measure of abdominal fat distribution, and increased WHR is a known predictor of metabolic dysfunction [30]. The data indicate a negative correlation between WHR and antipsychotic‐induced weight gain. It suggests that central adiposity, rather than overall weight gain, may be more closely linked to metabolic disruptions in patients taking AAs. Central obesity, characterized by an increased waist circumference relative to hip circumference, is associated with dyslipidaemia and insulin resistance [3]. The study by Li et al. [30] highlights that the distribution of weight gain may act as a significant factor in predicting metabolic changes in patients with schizophrenia treated with AAs. The case report by Wysokiński, A. and Sobów [31] showed a reduction of body weight once clozapine was stopped. The reversibility of these changes upon discontinuation of clozapine suggests that patients' lipid profiles and metabolic health may improve with appropriate adjustments to their treatment regimen. This provides a potential avenue for managing metabolic side effects in patients with schizophrenia.

### 4.6 Limitations

This review only included oral formulations of atypical antipsychotics. Long acting injectables (LAIs), which may have different pharmacokinetic properties affecting lipid metabolism, were not Represented in the Included studies. This may limit the generalizability of the findings to patients receiving LAI Treatment. Another limitation is the generalizability of the results. Many of the included studies analyzed data from patients of Chinese ethnicity. Genetic, dietary, and environmental Differences may influence metabolic responses to antipsychotics, potentially limiting the applicability of findings to non‐Asian populations. Future studies should aim to include more ethnically and geographically diverse populations.

Additionally, treatment duration varied considerably across the included studies, from short‐term interventions to long‐term follow‐ups. This variability may have influenced the lipid outcomes reported and introduces heterogeneity that limits the ability to draw firm conclusions. Existing research has suggested that the degree of adverse effects caused by AAs is dose‐dependent [10]. This scoping review did not focus on the dosage of AAs used. Furthermore, differences in antipsychotic dosage were noted but not systematically analyzed. Higher dosages may be associated with more pronounced lipid abnormalities. However, insufficient data on dosage‐response relationships restricted further exploration in this review.

Also, the included studies varied widely in design and sample size, ranging from case reports to large cohort studies. This methodological heterogeneity affects the strength and consistency of the conclusions drawn. This introduces variability in the reliability of findings. Additionally, sample sizes ranged from single‐patient reports to large cohort studies exceeding 600 participants, which may disproportionately influence interpretation. Smaller studies, particularly case reports, are more susceptible to selection bias and overestimation of effect sizes, while larger studies may offer more robust but less granular insights. This variation limits the ability to draw firm conclusions regarding the strength and consistency of associations between individual atypical antipsychotics and lipid abnormalities. Therefore, while the scoping review offers valuable insights, its findings should be interpreted with caution.

Another limitation is the potential confounding effect of illness severity on the observed relationship between specific AAs and lipid abnormalities. Clozapine and olanzapine are often reserved for treatment‐resistant or more severe cases of schizophrenia, while aripiprazole and other AAs may be prescribed in milder or early‐stage illness. These differences may reflect underlying variations in patient characteristics, including physical activity, dietary habits, and comorbidities such as diabetes or cardiovascular disease, which independently influence lipid metabolism. As a result, the metabolic outcomes attributed to specific AAs may, in part, reflect confounding by indication. Future studies should account for illness severity and lifestyle factors to better isolate the pharmacological impact of antipsychotics on lipid profiles.

This scoping review highlighted the adverse effects of individual AAs on lipid profile and possible associated complications including atherogenesis and hypertriglyceridaemia. However, this scoping review did not investigate the efficacy of different AAs on treating the positive and negative symptoms of schizophrenia. Therefore, it is difficult to recommend the use of a particular AA for the treatment of adults with schizophrenia. Indeed, it is recognized that antipsychotic prescribing should consider the risks and benefits for the individual patient [9].

## 5 Recommendations

Based on the findings of this scoping review, further research is needed to explore the link between AA use and developing dyslipidaemia. Firstly, the review identified a disparity in the number of studies analyzing AAs and dyslipidaemia. Whilst most of the studies included data on clozapine, olanzapine, risperidone, and aripiprazole, data on amisulpride, paliperidone, ziprasidone, and lurasidone was sparse. Therefore, more research is needed to facilitate more informed and personalized prescribing of AAs.

Additionally, more research is needed to compare the efficacy of individual AAs for treating symptoms of schizophrenia, alongside the incidence/prevalence of dyslipidaemia. This will facilitate clinicians and patients to weigh up the risks and benefits of different AAs when making prescribing choices.

Finally, the biomarkers identified in the study highlight potential therapeutic targets. For example, the association of the inflammatory biomarkers MIF and WBCs with AA‐induced dyslipidaemia, as well as the metabolic proteins asprosin, IGFBP‐2, and APOA1 highlight potential areas for further research.

## 6 Conclusion

This scoping review highlighted significant associations between specific atypical antipsychotics, particularly clozapine and olanzapine, and adverse lipid profiles in adult patients with schizophrenia. Analysis of the specific effects of AAs indicated that clozapine and olanzapine are more strongly associated with adverse impacts on the lipid profile, specifically characterized by elevated LDL and reduced HDL levels. In contrast, aripiprazole and lurasidone appear to exert potentially beneficial effects on dyslipidemia. Interestingly, several biological markers were identified, potentially highlighting their usefulness as monitoring parameters or therapeutic targets. Genetic polymorphisms, particularly those of the *APOA1* gene, were associated with a variation in the dyslipidaemic effect experienced by patients with schizophrenia treated with AAs. An awareness of this may influence prescribing choices in patients with known polymorphisms, as the field of pharmacogenetics and personalized treatment evolves.

Overall, this scoping review addressed the overarching research aim “to investigate and establish current understanding surrounding the association of atypical antipsychotics with lipid abnormalities in patients with schizophrenia.” This aids current understanding of the effect of individual AAs on developing dyslipidaemia, facilitates prescribing decisions, and highlights areas for future research including the effect of lesser studied AAs and potential biomarkers/therapeutic targets for AA‐induced dyslipidaemia.

## Author Contributions

Virginia Abavana read all articles and designed the PRISMA flow chart, analyzed relevant articles, wrote the whole manuscript, and checked references. Soban Sadiq designed the project, wrote the title, reviewed all articles, reviewed the analysis, and the whole manuscript.

## Consent

The authors have nothing to report.

## Conflicts of Interest

The authors declare no conflicts of interest.

## Supporting information


**Data S1:** Supporting Information.

## Data Availability

Data is available as Supporting Information in public repository figshare DOI: https://doi.org/10.6084/m9.figshare.29591594.v2.
