# Supplementary material for: Association of Atypical Antipsychotics With Lipid Abnormalities in Adult Patients With Schizophrenia: A Scoping Review
Source: Neuropsychopharmacol Rep. 2025 Sep 29;45(4):e70042. doi: 10.1002/npr2.70042 (PMC12477405; doi:10.1002/npr2.70042)
Supplement: Supplementary file 1 — Data S1: Supporting Information. [file NPR2-45-e70042-s001.docx]

### **The Preferred Reporting Items for Systematic reviews and Meta-Analyses extension for Scoping Reviews (PRISMA- ScR)**

| **SECTION** | **ITEM** | **PRISMA-ScR CHECKLIST ITEM** | **REPORTED ON PAGE #** |
| --- | --- | --- | --- |
| **TITLE** | | | |
| Title | 1 | Identify the report as a scoping review. | Click here to enter text. |
| **ABSTRACT** | | | |
| Structured summary | 2 | Provide a structured summary that includes (as applicable): background, objectives, eligibility criteria, sources of evidence, charting methods, results, and conclusions that relate to the review questions and objectives. | Click here to enter text. |
| **INTRODUCTION** | | | |
| Rationale | 3 | Describe the rationale for the review in the context of what is already known. Explain why the review questions/objectives lend themselves to a scoping review approach. | Click here to enter text. |
| Objectives | 4 | Provide an explicit statement of the questions and objectives being addressed with reference to their key elements (e.g., population or participants, concepts, and context) or other relevant key elements used to conceptualize the review questions and/or objectives. | Click here to enter text. |
| **METHODS** | | | |
| Protocol and registration | 5 | Indicate whether a review protocol exists; state if and where it can be accessed (e.g., a Web address); and if available, provide registration information, including the registration number. | N/A |
| Eligibility criteria | 6 | Specify characteristics of the sources of evidence used as eligibility criteria (e.g., years considered, language, and publication status), and provide a rationale. | Click here to enter text. |
| Information sources* | 7 | Describe all information sources in the search (e.g., databases with dates of coverage and contact with authors to identify additional sources), as well as the date the most recent search was executed. | Click here to enter text. |
| Search | 8 | Present the full electronic search strategy for at least 1 database, including any limits used, such that it could be repeated. | Click here to enter text. |
| Selection of sources of evidence† | 9 | State the process for selecting sources of evidence (i.e., screening and eligibility) included in the scoping review. | Click here to enter text. |
| Data charting process‡ | 10 | Describe the methods of charting data from the included sources of evidence (e.g., calibrated forms or forms that have been tested by the team before their use, and whether data charting was done independently or in duplicate) and any processes for obtaining and confirming data from investigators. | Click here to enter text. |
| Data items | 11 | List and define all variables for which data were sought and any assumptions and simplifications made. | Click here to enter text. |
| Critical appraisal of individual sources of evidence§ | 12 | If done, provide a rationale for conducting a critical appraisal of included sources of evidence; describe the methods used and how this information was used in any data synthesis (if appropriate). | Click here to enter text. |
| Synthesis of results | 13 | Describe the methods of handling and summarizing the data that were charted. | Click here to enter text. |
| **RESULTS** | | | |
| Selection of sources of evidence | 14 | Give numbers of sources of evidence screened, assessed for eligibility, and included in the review, with reasons for exclusions at each stage, ideally using a flow diagram. | Click here to enter text. |
| Characteristics of sources of evidence | 15 | For each source of evidence, present characteristics for which data were charted and provide the citations. | Click here to enter text. |
| Critical appraisal within sources of evidence | 16 | If done, present data on critical appraisal of included sources of evidence (see item 12). | Click here to enter text. |
| Results of individual sources of evidence | 17 | For each included source of evidence, present the relevant data that were charted that relate to the review questions and objectives. | Click here to enter text. |
| Synthesis of results | 18 | Summarize and/or present the charting results as they relate to the review questions and objectives. | Click here to enter text. |
| **DISCUSSION** | | | |
| Summary of evidence | 19 | Summarize the main results (including an overview of concepts, themes, and types of evidence available), link to the review questions and objectives, and consider the relevance to key groups. | Click here to enter text. |
| Limitations | 20 | Discuss the limitations of the scoping review process. | Click here to enter text. |
| Conclusions | 21 | Provide a general interpretation of the results with respect to the review questions and objectives, as well as potential implications and/or next steps. | Click here to enter text. |
| **FUNDING** | | | |
| Funding | 22 | Describe sources of funding for the included sources of evidence, as well as sources of funding for the scoping review. Describe the role of the funders of the scoping review. | N/A |

**A description of, and justification for each search term, alongside Boolean operators used.**

| **Search terms and Boolean operators used** |
| --- |
| ***#* 1 schizophrenia**  population of focus for scoping review as detailed in PICOT framework (Appendix 1).  ***#* 2 atypical antipsychotics OR second-generation antipsychotics**  intervention of focus for the scoping review as outlined in PICOT (Appendix 1). *Alternative terms provided to widen search, atypical and second-generation antipsychotics may both be used. Additionally, studies focusing on a broad range of antipsychotics may not specify which type.*  ***#* 3 lipid abnormalities OR dyslipidaemia OR dyslipidemia OR high cholesterol OR dyslipoproteinemia**  As detailed by the PICOT (Appendix 1), this is the outcome being analysed by this scoping review.  ***#* 4 *#1 AND #2 AND #3*** |

### **Table of final included papers.**

| First author (date) | Title |
| --- | --- |
| Vázquez-Bourgon (2022) | Aripiprazole and Risperidone Present Comparable Long-Term Metabolic Profiles: Data From a Pragmatic Randomized Controlled Trial in Drug-Naïve First-Episode Psychosis |
| Li (2018) | T 4 and waist:hip ratio as biomarkers of antipsychotic-induced weight gain in Han Chinese inpatients with schizophrenia |
| Wysokinski (2015) | Improvements in body composition,anthropometric measurements and lipid profile following discontinuation of clozapine |
| Wong (2024) | Pharmacogenetic Study of Antipsychotic–Induced Lipid and BMI Changes in Chinese Schizophrenia Patients: A Genome-Wide Association Study |
| Althanoon (2021) | Metabolic Adverse Effects of Antipsychotic Drugs in Patients with Schizophrenia |
| Kang (2015) | Metabolic Disturbances Independent of Body Mass in Patients with Schizophrenia Taking Atypical Antipsychotics |
| Amini (2024) | Effects of atypical antipsychotics on serum asprosin level and other metabolic parameters in patients with schizophrenia |
| Nolin (2020) | Circulating IGFBP-2 levels reveal atherogenic metabolic risk in schizophrenic patients using atypical antipsychotics |
| Correl (2016) | Long-term safety and effectiveness of lurasidone in schizophrenia: a 22-month, open-label extension study |
| Chen (2024) | High Circulating MIF Levels Indicate the Association with Atypical Antipsychotic-Induced Adverse Metabolic Effects |
| Fan (2021) | Association Between *APOA1* Gene Polymorphisms and Antipsychotic Drug-Induced Dyslipidemia in Schizophrenia |
| Xiong (2019) | Association of blood cell counts with the risk of olanzapine‐ or clozapine‐induced dyslipidemia in Chinese schizophrenia patients |

### **Charting Table used to gather data from selected studies.**

| First author, year of publication | Study design | Objective | Participants (n) and demographics | Main findings | Effect of individual AAs on lipid profile |
| --- | --- | --- | --- | --- | --- |
| Vázquez-Bourgon (2022) | Randomised controlled trial | To assess the long term metabolic profile of drug-naïve adult patients with schizophrenia, treated with aripiprazole or risperidone | -n=188  Adult patients  First episode non-affective schizophrenia | Both Aripiprazole and Risperidone showed similar long-term metabolic profiles | Total Cholesterol: Both drugs showed a small increase in total cholesterol, but this change was not statistically significant between aripiprazole and risperidone.  LDL: Risperidone was associated with a slightly higher increase in LDL compared to aripiprazole.  HDL: There was a small reduction in HDL cholesterol in both groups, but this was not significantly different between the two drugs.  Triglycerides: Both aripiprazole and risperidone were linked to modest increases in triglyceride levels, with no significant differences between them |
| Li (2018) | Cross-sectional  prospective, observational study | To assess the use of T4 and Waist to hip ratio as predictors for weight gain in patients treated with AAs. | -n=264  Chinese  Adults  Schizophrenia  Inpatients | Higher levels of T4 and a higher waist-to-hip ratio were associated with increased weight gain during treatment with atypical antipsychotics. Olanzapine showed a more significant increase compared to risperidone | Olanzapine was linked to significantly higher increase in LDL levels over 12 weeks, compared to risperidone. |
| Wysokinski (2015) | Case report | To demonstrate a case in which a patient treated with AAs developed severe triglyceridemia, and the consequences of this | -n=1  Male  White european | Association of clozapine with the development of a severe triglyceridemia  32 year old male patient experienced clozapine-induced severe triglyceridaemia.  TG, TC and LDL decreased considerably. HDL showed a small increase once clozapine withdrawn. |  |
| Wong (2024) | Longitudinal cohort | To identify genetic variants associated with metabolic side effects—specifically lipid profile alterations and changes in body mass index (BMI)—induced by AAs. | -n=625  -Adults  -Chinese ethnicity  -Treated with AAs  -At least 1 post-AA measurement of fasting lipids +/- BMI  -no pre-existing metabolic disorders | Identified single nucleotide polymorphisms (SNPs) significantly associated with antipsychotic-induced metabolic changes, including: rs6532055 in ABCG2, rs2644520 near SORCS1, rs115843863 near UPP2, rs2514895 near KIRREL3, and rs188405603 in SLC2A9.  Additionally, gene-based analyses highlighted six genes—ABCG2, APOA5, ZPR1, GCNT4, MAST2, and CRTAC1—as significantly linked to these metabolic side effects. | Olanzapine-induced LDL changes: The SNP rs6532055 in the ABCG2 gene was significantly associated.​  Aripiprazole-induced triglyceride changes: The SNP rs2644520, located near the SORCS1 gene, showed a significant association.​  Clozapine-induced HDL changes: The SNP rs115843863, near the UPP2 gene, was significantly linked.​  Paliperidone-induced LDL changes: The SNP rs2514895, near the KIRREL3 gene, demonstrated a significant association.​  Quetiapine-induced triglyceride changes: The SNP rs188405603 in the SLC2A9 gene was significantly associated. |
| Althanoon (2021) | Cohort study | To compare the efficacy and metabolic tolerability of aripiprazole and olanzapine in adults with schizophrenia. | -n=80  Schizophrenia  Antipsychotic -free 6 months | Positive correlation between BMI and Leptin | Olanzapine:​  Induced significant increases in triglycerides, LDL-C, and total cholesterol levels, alongside a decrease in HDL-C.​  Aripiprazole:​  Led to minor, statistically insignificant increases in triglycerides and LDL-C, with no significant effect on HDL-C. |
| Kang (2015) | Observational, cross-sectional study | To understand the role of atypical antipsychotics in the development of metabolic disturbances, regardless of the patients' body weight. | -n=174 | Patients with schizophrenia taking atypical antipsychotics showed metabolic disturbances, including changes in lipid profiles, even after controlling for BMI.  Lipid Profile Changes:  Atypical antipsychotics were found to have significant effects on lipid metabolism, independent of weight gain. Observed increases in total cholesterol and triglycerides and decreases in HDL.  Clozapine: significant metabolic disturbances, including increases in total cholesterol, LDL cholesterol, and triglycerides, even in the absence of substantial weight gain.  Olanzapine had a strong association with increased triglyceride levels and a decrease in HDL cholesterol. It also led to increases in total cholesterol and LDL cholesterol.  Risperidone also had significant effects on lipid metabolism, though its impact was less compared to olanzapine. Risperidone was linked to an increase in triglycerides and a modest decrease in HDL cholesterol.  Quetiapine showed moderate effects on lipid metabolism, with some increase in triglycerides and total cholesterol, but its effects were less severe compared to olanzapine.  Aripiprazole did not have a significant impact on lipid profiles.  Ziprasidone: had a milder effect on lipid profiles, with only slight increases in total cholesterol and triglycerides, and had minimal impact on LDL and HDL. | |
| Amini (2024) | Observational, cross-sectional study. | To evaluate the effects of atypical antipsychotics on serum asprosin levels (a protein related to metabolism and appetite regulation) and other metabolic parameters, such as lipid profiles, blood glucose levels, and insulin resistance. | -n=62 | Asprosin levels were significantly elevated in these patients treated with AAs, compared to healthy controls  High serum asprosin levels were associated with metabolic disturbances, including insulin resistance and dyslipidemia. | Olanzapine treatment was associated with increased levels of total cholesterol, triglycerides, and LDL (low-density lipoprotein), while HDL (high-density lipoprotein)  Risperidone also had notable effects on lipid metabolism, leading to increased triglyceride levels and a decrease in HDL cholesterol. However, its effects on total cholesterol and LDL were less pronounced compared to olanzapine.  Aripiprazole had a more favorable lipid profile compared to the other atypical antipsychotics. It had minimal impact on triglycerides, total cholesterol, and LDL, and did not significantly alter HDL levels. |
| Nolin (2020) | Observational, cross-sectional study | To assess how atypical antipsychotics influence IGFBP-2 levels (a biomarker related to metabolic health) and lipid profiles (e.g., cholesterol, triglycerides, LDL, HDL) in patients with schizophrenia. | -n=87  Schizophrenia  Men  Mean treatment duration 20 months | Patients treated with atypical antipsychotics had elevated serum IGFBP-2 levels, which were associated with a higher risk of atherogenic metabolic disturbances.  Elevated IGFBP-2 levels were found to be a potential biomarker for predicting atherogenic metabolic risk, suggesting that these patients may be at higher risk for cardiovascular diseases due to the metabolic disturbances induced by their medications.  patients on atypical antipsychotics had dyslipidemia, including increased levels of total cholesterol, triglycerides, and LDL cholesterol, along with decreased levels of HDL cholesterol, leading to an atherogenic lipid profile.  These changes were found to be independent of (BMI), | Olanzapine and risperidone caused significant lipid disturbances  Olanzapine treated patients showed a higher cholesterol/HDL ratio, and higher levels of triglycerides compared to patients treated with risperidone |
| Correl (2016) | Open-label extension study. | To assess the long-term safety and effectiveness of lurasidone in managing schizophrenia, with a focus on its impact on psychiatric symptoms, quality of life, and metabolic parameters such as lipid profile, weight, and glucose levels. | Schizophrenia  Treated with lurasidone for 22 months | Lurasidone:  Minimal Impact on Lipid Profiles: Lurasidone had no significant changes in total cholesterol, LDL, HDL, or triglycerides over the long term. This finding indicates that lurasidone does not significantly worsen lipid metabolism, which is a considerable advantage over many other atypical antipsychotics.  Compared to other antipsychotics, lurasidone is associated with a low risk of metabolic disturbances, making it a preferable option for patients who are at risk of cardiovascular or metabolic issues. | |
| Chen (2024) | Cross-sectional | To investigate the relationship between circulating MIF levels and metabolic disturbances (including lipid profile alterations, insulin resistance, and weight gain) in patients with schizophrenia treated with atypical antipsychotics. | -n=150  Schizophrenia  Diagnosed by DSM-5 | Patients receiving atypical antipsychotics had significantly higher circulating MIF levels compared to healthy controls.  Elevated MIF levels were positively correlated with adverse metabolic effects, including dyslipidemia, insulin resistance, and weight gain.  Metabolic Disturbances:  The results indicated that high MIF levels were associated with the following changes in lipid profiles:  Increased total cholesterol and LDL cholesterol.  Decreased HDL cholesterol (which is considered "good" cholesterol).  Increased triglyceride levels, indicating an atherogenic lipid profile.  Insulin Resistance and Weight Gain:  Elevated MIF levels were associated with increased insulin resistance and significant weight gain in patients on atypical antipsychotics.  These metabolic disturbances were linked to a higher risk of developing cardiovascular diseases. | Olanzapine had the most significant impact on lipid profiles, with increased total cholesterol, LDL, and triglycerides, and decreased HDL, all of which were correlated with higher MIF levels.  Risperidone and quetiapine also caused lipid profile changes (increased TChol, LDL and decreased HDL), but the effects were less pronounced than with olanzapine.  Aripiprazole had a more favorable metabolic profile, with minimal effects on lipids and lower MIF levels compared to the other atypical antipsychotics. |
| Fan (2021) | Case-control study | To investigate the relationship between *APOA1* gene polymorphisms and antipsychotic drug-induced dyslipidemia in patients with schizophrenia. Specifically, the study sought to identify if genetic variations in APOA1 were associated with lipid profile disturbances in patients treated with atypical antipsychotics. | -n=600  300 in treatment arm  300 healthy controls | Main Findings  *APOA1* Gene Polymorphisms and Dyslipidemia  *APOA1* gene polymorphisms (*APOA1* −75 G/A and *APOA1* 83C/T variants) were significantly associated with higher risk of dyslipidemia in patients treated with atypical antipsychotics.  Linked to alterations in lipid profiles, such as elevated total cholesterol, increased triglycerides, and decreased HDL cholesterol.  The *APOA1* −75 G/A polymorphism was associated with higher triglyceride levels and lower HDL levels in patients on atypical antipsychotics.  The *APOA1* 83C/T polymorphism was also correlated with higher total cholesterol and LDL cholesterol levels in patients treated with antipsychotics.  The *APOA1* gene plays a role in modulating lipid metabolism, and certain polymorphisms in this gene may contribute to the severity of dyslipidemia induced by atypical antipsychotics.   - *APOA1*gene polymorphisms (particularly −75 G/A and 83C/T) were found to be associated with more severe dyslipidemia in patients treated with atypical antipsychotics. - Olanzapine was the most lipid-altering among the atypical antipsychotics, significantly increasing total cholesterol, LDL, and triglycerides, and decreasing HDL. - Risperidone and quetiapine also caused lipid changes but were less potent than olanzapine in altering lipid profiles. - Aripiprazole had a more neutral effect on lipid profiles, with minimal changes in total cholesterol, LDL, HDL, and triglycerides.   The findings suggest that genetic testing for *APOA1* polymorphisms could be useful in predicting antipsychotic-induced dyslipidemia and personalizing treatment to minimize metabolic side effects, particularly in patients using medications like olanzapine. | Olanzapine:  Total Cholesterol: Olanzapine treatment led to a significant increase in total cholesterol levels.  LDL cholesterol levels were notably increased in patients treated with olanzapine.  HDL Olanzapine caused a decrease in HDL cholesterol, which is a negative effect on lipid metabolism.  Triglyceride levels were also elevated with olanzapine treatment, leading to an atherogenic lipid profile.  Risperidone:  Total Cholesterol: Risperidone also caused an increase in total cholesterol.  LDL cholesterol was elevated, though to a lesser extent than with olanzapine.  HDL: Risperidone treatment led to a decrease in HDL cholesterol.  Triglyceride levels were moderately increased with risperidone.  Quetiapine  Total Cholesterol: Quetiapine resulted in a moderate increase in total cholesterol levels.  LDL cholesterol was slightly elevated in patients on quetiapine.  HDL cholesterol levels were generally decreased with quetiapine treatment.  Triglyceride levels were slightly increased with quetiapine.  Aripiprazole:  Total Cholesterol: Aripiprazole had minimal effects on total cholesterol.  LDL cholesterol showed little to no change with aripiprazole.  HDL cholesterol remained stable with aripiprazole treatment.  Triglyceride levels remained unchanged with aripiprazole, making it one of the atypical antipsychotics with the least impact on lipid profiles. |
| Xiong (2019) | Retrospective, observational cohort study | To investigate the relationship between blood cell counts and the risk of dyslipidemia induced by olanzapine and clozapine in Chinese schizophrenia patients | n=120  Chinese ethnicity  Schizophrenia | Association Between Blood Cell Counts and Dyslipidemia:  Increased white blood cell (WBC) counts and platelet counts were significantly associated with an increased risk of developing dyslipidemia in patients treated with olanzapine or clozapine.  Higher WBC counts were linked to elevated triglyceride levels  Increased platelet counts were associated with higher LDL cholesterol and lower HDL cholesterol.  Red Blood Cells (RBC): Red blood cell counts were less frequently associated with dyslipidemia | - Olanzapine had the most pronounced negative effects on lipid profiles, leading to elevated total cholesterol, LDL, triglycerides, and decreased HDL cholesterol. - Clozapine also caused dyslipidemia, but the lipid changes were generally less severe than those seen with olanzapine. |

### **Critical Appraisal of cohort studies based on Critical Appraisal Skills Programme (CASP) tool for cohort studies**

| Pharmacogenetic Study of Antipsychotic–Induced Lipid and BMI Changes in Chinese Schizophrenia Patients: A Genome-Wide Association Study, Wong (2024) |
| --- |
| Did the study address a clearly focused issue?  Answer: Yes. The study focused on the genetic factors that influence lipid and BMI changes in Chinese schizophrenia patients treated with antipsychotics. It specifically examined how genetic variations (e.g., single nucleotide polymorphisms) may contribute to metabolic changes induced by antipsychotic medications.  2. Was the cohort recruited in an acceptable way?  Answer: Yes. The cohort consisted of Chinese schizophrenia patients, and if selection criteria were well-defined (e.g., diagnosis confirmation, exclusion of comorbidities).  3. Was the exposure accurately measured to minimize bias?  Answer: Yes. The study analysed dose and type of antipsychotic treatment and the corresponding doses, alongside any concomitant statin therapy or antidepressants.  4. Was the outcome accurately measured to minimize bias?  Answer: Yes. Standard lipid profiles and BMI measurements were used. These measurements were taken annually during treatment with antipsychotics.  5. (a) Have the authors identified all important confounding factors?  Answer: Yes, treatment type, and baseline metabolic status were key confounders. However, lifestyle factors (e.g., diet, physical activity), comorbidities, and other medications might be important confounders that could influence lipid or BMI changes.  (b) Have they taken account of the confounding factors in the design and/or analysis?  Answer: Yes, they used ‘within-subject’ effects to account for time-invariant confounders.  6. (a) Was the follow-up of subjects complete enough?  Answer: Yes, 5.7 years median follow up.  (b) Was the follow-up of subjects long enough?  Answer: Yes, 5.7 years median follow up.  7. What are the results of this study?  Answer: The study identified specific genetic variants/SNPs that were associated with lipid and BMI changes in schizophrenia patients on antipsychotics.  8. How precise are the results?  Answer: Six genes reached the genome-wide analyses threshold for statistical significance. Confounders were accounted for in the statistical analyses.  9. Do you believe the results?  Answer: Yes, large population with appropriate statistical analyses.  10. Can the results be applied to the local population?  Answer: Yes, given that the study was conducted in a Chinese population, the results are directly applicable to schizophrenia patients in China. However, findings may not generalise to other ethnic groups without further studies.  11. Do the results of this study fit with other available evidence?  Answer: Yes  12. What are the implications of this study for practice?  Answer: The specific genetic markers have been found to predict metabolic side effects, so this could lead to more personalized treatments, allowing clinicians to select antipsychotics with fewer metabolic side effects based on individual genetic profiles. |
| Metabolic Adverse Effects of Antipsychotic Drugs in Patients with Schizophrenia, Althanoon (2021) |
| 1. Did the study address a clearly focused issue?  Answer: Yes. The study aimed to evaluate the metabolic side effects (e.g., lipid abnormalities, weight gain) associated with various antipsychotic drugs in schizophrenia patients.  2. Was the cohort recruited in an acceptable way?  Answer: Yes.The cohort was recruited from schizophrenia inpatients and outpatients, with well-defined criteria were for selecting participants (DSM-V5).  3. Was the exposure accurately measured to minimize bias?  Answer: Yes, antipsychotic treatment type and doses were considered, with consideration for patient adherence.  4. Was the outcome accurately measured to minimize bias?  Answer: Yes, the study likely measured standard parameters of lipid profile and BMI. These were repeated before and after 6 months of treatment.  5. (a) Have the authors identified all important confounding factors?  Answer: The authors likely considered medication dose, duration, and type, but lifestyle factors such as diet, physical activity, and comorbidities may also play a role in metabolic effects and might need to be considered.  (b) Have they taken account of the confounding factors in the design and/or analysis?  Answer: This is uncertain without more information, but ideally, the study should control for lifestyle and other factors through multivariate analysis.  6. (a) Was the follow-up of subjects complete enough?  Answer: Likely yes, assuming the study had sufficient duration to detect changes in lipid and BMI outcomes. A follow-up of at least 6 months would be expected for observing metabolic changes.  (b) Was the follow-up of subjects long enough?  Answer: Yes, the follow-up was 6 months.  7. What are the results of this study?  Answer: The study demonstrated that certain antipsychotics (e.g., olanzapine) are associated with more pronounced metabolic changes (e.g., weight gain, lipid abnormalities) than others (e.g., aripiprazole).  8. How precise are the results?  Answer: Statistical significance (p<0.01) for increase in total cholesterol and triglycerides following treatment with olanzapine, but not aripiprazole.  9. Do you believe the results?  Answer: Yes, the study followed appropriate methods. Although, it is not fully clear how confounders were taken into account.  10. Can the results be applied to the local population?  Answer: Yes, the study’s findings would be applicable to schizophrenia patients treated with antipsychotics in the same region.  11. Do the results of this study fit with other available evidence?  Answer: Yes, the findings are consistent with existing literature, which shows metabolic side effects with antipsychotics, particularly those like olanzapine.  12. What are the implications of this study for practice?  Answer: The study reinforces the need to monitor metabolic side effects, particularly with certain antipsychotics, and suggests that clinicians consider these risks when prescribing antipsychotics. |
| Association of Blood Cell Counts with the Risk of Olanzapine‐ or Clozapine‐Induced Dyslipidemia in Chinese Schizophrenia Patients, Xiong (2019) |
| 1. Did the study address a clearly focused issue?  Yes. This study examined the association between blood cell counts and lipid abnormalities induced by olanzapine and clozapine in schizophrenia patients.  2. Was the cohort recruited in an acceptable way?  Yes, patients with schizophrenia were appropriately selected and diagnosed, and inclusion/exclusion criteria were clearly defined.  3. Was the exposure accurately measured to minimize bias?  Answer: Yes, the exposure was well-defined. Blood cell counts were measured before and after 2-4 weeks of treatment.  4. Was the outcome accurately measured to minimize bias?  Answer: Yes, lipid profiles were likely measured using standard laboratory tests. Blood cell counts were also measured using standard parameters.  5. (a) Have the authors identified all important confounding factors?  Answer: The study considered medication type and dose. Gender was taken into account.  (b) Have they taken account of the confounding factors in the design and/or analysis?  Answer: Yes, they have divided the results into male and female, but it is unclear how lifestyle factors/other medications were taken into account.  6. (a) Was the follow-up of subjects complete enough?  Answer: The follow up was short-term, 2-4 weeks.  (b) Was the follow-up of subjects long enough?  Answer: Short-term follow up- after 2-4 weeks. So conclusions can be drawn for short-term effect.  7. What are the results of this study?  Answer: The study found that blood cell counts, such as white blood cells and platelets are associated with lipid abnormalities in patients treated with olanzapine and clozapine.  8. How precise are the results?  Answer: A large sample size was used and statistical methods were rigorous.  9. Do you believe the results?  Answer: Yes, the study used appropriate methods, and main conclusions were based on statistically significant results.  10. Can the results be applied to the local population?  Answer: Yes, as the study was conducted in Chinese schizophrenia patients, the results are most directly applicable to this population.  11. Do the results of this study fit with other available evidence?  Answer: Yes, the findings are consistent with literature suggesting that antipsychotics like olanzapine and clozapine are associated with dyslipidemia, possibly mediated by changes in blood cell counts.  12. What are the implications of this study for practice?  Answer: The study suggests that monitoring blood cell counts in patients on olanzapine or clozapine could help identify those at risk of metabolic abnormalities, prompting early intervention. |
|  |

### **Critical Appraisal of cross-sectional studies based on Critical Appraisal Skills Programme (CASP) tool for cross-sectional studies**

| T4 and Waist:Hip Ratio as Biomarkers of Antipsychotic-Induced Weight Gain in Han Chinese Inpatients with Schizophrenia, Li (2018) |
| --- |
| 1. Did the study address a clearly focused issue?  Answer: Yes. The study focused on the relationship between T4 (thyroxine) and waist-to-hip ratio (WHR) as biomarkers for antipsychotic-induced weight gain in Han Chinese inpatients with schizophrenia.  2. Did the authors use an appropriate method to answer their question?  Answer: Yes. The study measured T4 levels and WHR and compared them with changes in body weight following antipsychotic treatment.  3. Were the subjects recruited in an acceptable way?  Answer: Yes. The study involved inpatients with schizophrenia, specifically Han Chinese individuals. There were clear inclusion and exclusion criteria.  4. Were the measures accurately measured to reduce bias?  Answer: Yes. T4 levels and waist-to-hip ratios are standard, objective measures.  5. Were the data collected in a way that addressed the research issue?  Answer: Yes. The study aimed to explore metabolic changes, and measuring thyroid hormone levels and body composition (via waist-to-hip ratio) would directly address the research issue of metabolic side effects in schizophrenia patients treated with antipsychotics.  6. Did the study have enough participants to minimize the play of chance?  Answer: Yes, 296 patients were recruited.  7. How are the results presented and what is the main result?  Answer: Results are presented in terms of correlations between T4 levels, WHR, and weight gain in schizophrenia patients on antipsychotics. The main result is that there was a correlation between these biomarkers and antipsychotic-induced metabolic changes.  8. Was the data analysis sufficiently rigorous?  Answer: Yes, regression analysis was used to assess the relationship between biomarkers and weight gain.  9. Is there a clear statement of findings?  Answer: Yes. The study clearly states that T4 and WHR are significant biomarkers for weight gain due to antipsychotic treatment in this patient population.  10. Can the results be applied to the local population?  Answer: Yes, the study specifically focused on Han Chinese inpatients, so the results are most applicable to this population. However, generalizing beyond this group requires further studies in other populations.  11. How valuable is the research?  Positive/Methodologically sound aspects: The study’s focus on identifying biomarkers for antipsychotic-induced weight gain is valuable, particularly for tailored treatments in schizophrenia.  Negative/Relatively poor methodology: The study may lack control for other confounding factors (e.g., lifestyle, diet, other medications).  Unknowns: Exact confounders taken into account. |
| Metabolic Disturbances Independent of Body Mass in Patients with Schizophrenia Taking Atypical Antipsychotics, Kang (2015) |
| 1. Did the study address a clearly focused issue?  Answer: Yes. The study aimed to investigate metabolic disturbances in schizophrenia patients treated with atypical antipsychotics, independent of changes in body mass.  2. Did the authors use an appropriate method to answer their question?  Answer: Yes. The study measured various metabolic parameters (e.g., lipids, insulin resistance) alongside weight gain to differentiate metabolic disturbances due to antipsychotics from those due to body mass changes.  3. Were the subjects recruited in an acceptable way?  Answer: Yes. The study included schizophrenia patients on atypical antipsychotics, and used clear inclusion/exclusion criteria.  4. Were the measures accurately measured to reduce bias?  Answer: Yes. Metabolic measures such as lipid levels, insulin, and body mass index (BMI) were measured using standard methods.  5. Were the data collected in a way that addressed the research issue?  Answer: Yes. The study measured both metabolic markers and body mass separately to isolate the metabolic disturbances caused by antipsychotics, independent of weight gain.  6. Did the study have enough participants to minimize the play of chance?  Answer: Yes, there were 174 participants.  7. How are the results presented and what is the main result?  Answer: Yes, results were presented clearly in text and tables. The main result is that metabolic disturbances are significant after AA use, even without affecting BMI.  8. Was the data analysis sufficiently rigorous?  Answer: Yes, they did multivariate regression to account for confounders and isolate the effect of the antipsychotic drugs.  9. Is there a clear statement of findings?  Answer: Yes. There was a clear statement suggesting that disturbances (e.g., dyslipidemia, insulin resistance) can occur independently of body weight changes in patients taking atypical antipsychotics.  10. Can the results be applied to the local population?  Answer: Yes.  11. How valuable is the research?  Answer: Positive/Methodologically sound aspects: The focus on metabolic disturbances independent of weight gain is highly valuable for improving understanding of antipsychotic side effects.  Negative/Relatively poor methodology: The study may have limited generalizability if it doesn’t account for all potential confounders (e.g., diet, exercise), also it only focuses on patients from Han Chinese ethinicity.  Unknowns: -- |
| Circulating IGFBP-2 Levels Reveal Atherogenic Metabolic Risk in Schizophrenic Patients Using Atypical Antipsychotics, Nolin (2020) |
| 1. Did the study address a clearly focused issue?  Answer: Yes. The study addressed the role of circulating IGFBP-2 (insulin-like growth factor-binding protein 2) as a potential marker for atherogenic metabolic risk in schizophrenia patients on atypical antipsychotics.  2. Did the authors use an appropriate method to answer their question?  Answer: Yes. Measuring circulating IGFBP-2 levels and linking them to metabolic markers like lipids or insulin resistance would be an appropriate method for exploring the relationship between IGFBP-2 and metabolic risk in schizophrenia.  3. Were the subjects recruited in an acceptable way?  Answer: Yes, patients with schizophrenia on atypical antipsychotics were selected using appropriate inclusion/exclusion criteria.  4. Were the measures accurately measured to reduce bias?  Answer: Yes. IGFBP-2 levels are likely measured using standard, reliable laboratory methods.  5. Were the data collected in a way that addressed the research issue?  Answer: Yes, the study measured both IGFBP-2 and relevant metabolic outcomes (lipid profiles, insulin levels), this would directly address the research issue.  6. Did the study have enough participants to minimize the play of chance?  Answer: Yes, the study focused on 174 patients.  7. How are the results presented and what is the main result?  Answer: The results are presented in terms of the association between IGFBP-2 levels and atherogenic metabolic risk markers like LDL, triglycerides, and insulin resistance. The main result is a significant relationship between elevated IGFBP-2 and metabolic abnormalities in schizophrenia patients using atypical antipsychotics.  8. Was the data analysis sufficiently rigorous?  Answer: Yes, logistic regression was used for confounders.  9. Is there a clear statement of findings?  Answer: Yes. The study concludes that elevated IGFBP-2 levels are associated with increased atherogenic risk in schizophrenia patients on atypical antipsychotics.  10. Can the results be applied to the local population?  Answer: Yes, the study focused on Chinese schizophrenia patients on antipsychotics, the results would be most applicable to this group. However, further studies in other populations would be necessary for broader applicability.  11. How valuable is the research?  : Positive/Methodologically sound aspects: The research could be valuable in identifying a novel biomarker (IGFBP-2) for metabolic risk in antipsychotic-treated schizophrenia patients.  Negative/Relatively poor methodology: The study may not account for all potential confounding variables (e.g., diet, exercise).  Unknowns: - |
| Effects of Atypical Antipsychotics on Serum Asprosin Level and Other Metabolic Parameters in Patients with Schizophrenia, Amini (2024) |
| 1. Did the study address a clearly focused issue?  Answer: Yes. The study focused on the effect of atypical antipsychotics on serum asprosin levels and other metabolic parameters in patients with schizophrenia.  2. Did the authors use an appropriate method to answer their question?  Answer: Yes. The study measured asprosin levels along with standard metabolic markers such as blood glucose, insulin resistance, and lipid profiles.  3. Were the subjects recruited in an acceptable way?  Answer: Yes. The study recruited schizophrenia patients on atypical antipsychotics using clear inclusion/exclusion criteria.  4. Were the measures accurately measured to reduce bias?  Answer: Yes. Asprosin levels and metabolic markers like glucose and lipid profiles were measured using accurate, standardised laboratory methods.  5. Were the data collected in a way that addressed the research issue?  Answer: Yes. By assessing both serum asprosin levels and metabolic parameters in patients treated with atypical antipsychotics, the study would directly address its research question about how these medications influence metabolic function.  6. Did the study have enough participants to minimize the play of chance?  Answer: The study only had 62 participants.  7. How are the results presented and what is the main result?  Answer: Results were presented as comparisons between pre- and post-treatment asprosin levels and metabolic parameters (e.g., lipids, glucose). The main result was that asprosin levels had a statistically significant association with blood glucose and triglyceride levels in patients treated with AAs.  8. Was the data analysis sufficiently rigorous?  Answer: Not clear how confounders were accounted for in statistical analyses.  9. Is there a clear statement of findings?  Answer: Yes. The study provided a clear statement regarding the association between atypical antipsychotics, asprosin levels, and metabolic abnormalities in schizophrenia patients.  10. Can the results be applied to the local population?  Answer: The results are applicable to the Iranian population. Further studies would be needed to confirm generalizability to other populations.  11. How valuable is the research?  Answer: Positive/Methodologically sound aspects: The research explores the potential role of asprosin as a biomarker for antipsychotic-induced metabolic disturbances, which is valuable for personalized treatment strategies.  Negative/Relatively poor methodology: The study lacks detailed information on confounders (e.g., diet, exercise), which could influence metabolic outcomes.  Unknowns: Sample size and follow-up duration are not provided, which limits the ability to assess the reliability and generalizability of the findings. |
| High Circulating MIF Levels Indicate the Association with Atypical Antipsychotic-Induced Adverse Metabolic Effects, Chen (2024) |
| 1. Did the study address a clearly focused issue?  Answer: Yes. The study focuses on the role of high circulating MIF (macrophage migration inhibitory factor) levels in mediating adverse metabolic effects in patients with schizophrenia treated with atypical antipsychotics.  2. Did the authors use an appropriate method to answer their question?  Answer: Yes. If the study measured MIF levels alongside metabolic parameters such inlcuding blood glucose, insulin resistance, or lipid profiles, then it used an appropriate method to investigate the relationship between MIF and metabolic disturbances caused by antipsychotic use.  3. Were the subjects recruited in an acceptable way?  Answer: Yes. The study included patients diagnosed with schizophrenia who were on atypical antipsychotic medications, with specific inclusion/exclusion criteria.  4. Were the measures accurately measured to reduce bias?  Answer: Yes. MIF levels and metabolic parameters are measurable using well-established laboratory techniques (ELISA for MIF, lipid panels for cholesterol).  5. Were the data collected in a way that addressed the research issue?  Answer: Yes. The collection of both MIF levels and metabolic markers (lipids, glucose) in schizophrenia patients treated with atypical antipsychotics directly addresses the research issue.  6. Did the study have enough participants to minimize the play of chance?  Answer: Yes, 142 participants.  7. How are the results presented and what is the main result?  Answer: Results were presented in terms of the correlation between high MIF levels and adverse metabolic effects in patients on atypical antipsychotics. The main result highlighted a significant association between elevated MIF levels and conditions like dyslipidemia, insulin resistance, or weight gain.  8. Was the data analysis sufficiently rigorous?  Answer: Yes, multiple linear regression analyses were used.  9. Is there a clear statement of findings?  Answer: Yes. The study clearly stated that high MIF levels are significantly associated with metabolic disturbances in schizophrenia patients treated with AAs  10. Can the results be applied to the local population?  Answer: Yes, the results are applicable to Han Chinese population. Additional studies would be needed to confirm whether the findings generalise to other populations.  11. How valuable is the research?  Answer: Positive/Methodologically sound aspects: The study provides valuable insight into the potential role of MIF as a biomarker for atypical antipsychotic-induced metabolic disturbances, which could lead to better management strategies for affected patients.  Negative/Relatively poor methodology: Follow up duration unclear  Unknowns: Follow up duration unclear. |

### **Critical appraisal of RCT using CASP tool for RCTs**

| Aripiprazole and Risperidone Present Comparable Long-Term Metabolic Profiles: Data From a Pragmatic Randomized Controlled Trial in Drug-Naïve First-Episode Psychosis, Vázquez-Bourgon, J (2022) |
| --- |
| 1. Did the study address a clearly formulated research question?  Answer: Yes. The research question is clearly formulated: "Are the long-term metabolic profiles of aripiprazole and risperidone comparable in drug-naïve first-episode psychosis patients?" The focus is on comparing the metabolic effects (e.g., weight gain, lipid profiles, insulin resistance) of two antipsychotic medications over time in a controlled setting.  2. Was the assignment of participants to interventions randomized?  Answer: Yes. The participants were randomly assigned to receive either aripiprazole or risperidone.  3. Were all participants who entered the study accounted for at its conclusion?  Answer: Yes, the RCT reported on participant retention and drop-out rates.  4. (a) Were the participants ‘blind’ to the intervention they were given?  Answer: Yes  (b) Were the investigators ‘blind’ to the intervention they were giving to participants?    Answer: No  (c) Were the people assessing/analysing outcomes ‘blinded’?  Answer: Yes  5. Were the study groups similar at the start of the randomized controlled trial?  Answer: Yes, groups are similar at baseline: age, gender, and initial metabolic status, drug-naivety.  6. Apart from the experimental intervention, did each study group receive the same level of care (that is, were they treated equally)?  Answer: Yes, the monitoring parameters were measured at the same intervals.  7. Were the effects of the intervention reported comprehensively?  Answer: Yes, the effects were reported comprehensively, including for primary metabolic outcomes (weight gain, insulin resistance, lipid profile) and follow up duration.  8. Was the precision of the estimate of the intervention or treatment effect reported?  Answer: Yes.  9. Do the benefits of the experimental intervention outweigh the harms and costs?  Answer: Yes, both medications had comparable metabolic profiles with minimal adverse effects, the benefits may outweigh the harms.  10. Can the results be applied to your local population/in your context?  Answer: Yes, if the findings are applicable to drug-naïve first-episode psychosis patients. But study was carried out in Spain. in the study is similar to your local population.  11. Would the experimental intervention provide greater value to the people in your care than any of the existing interventions?  Answer: If aripiprazole and risperidone present comparable long-term metabolic profiles, the choice between the two medications may depend on other factors, such as efficacy for psychotic symptoms, side effect profiles, and cost. Eg., if drug has fewer side effects in the context of schizophrenia treatment, it could provide greater overall value. |
| SUMMARY  Positive/Methodologically Sound Aspects:  Randomized Controlled Trial Design: Gold standard design that minimizes bias and ensures valid comparisons.  Clear Research Question: The study addresses an important and clear question about the long-term metabolic effects of two commonly used antipsychotics in first-episode psychosis patients.  Comprehensive Outcome Reporting: The study reports on a broad range of metabolic outcomes, making the findings useful for clinical decision-making.  Negative/Relatively Poor Methodology:  Blinding Issues: While participants are likely blinded, investigator blinding may not always be possible when administering medications. This could introduce bias, particularly in subjective assessments.  Unknown Aspects:  Follow-up Duration: The study should report whether the follow-up period was long enough to observe meaningful long-term metabolic effects. |
|  |
|  |

### **Critical appraisal for case-control study based on CASP appraisal tool.**

| Association Between *APOA1* Gene Polymorphisms and Antipsychotic Drug-Induced Dyslipidemia in Schizophrenia (Fan, 2021) |
| --- |
| 1. Did the study address a clearly focused issue?  Answer: Yes. The study addresses a clearly focused issue: the association between *APOA1* gene polymorphisms and antipsychotic drug-induced dyslipidemia in patients with schizophrenia.  2. Did the authors use an appropriate method to answer their question?  Answer: Yes. The study used a case-control design to assess the relationship between specific genetic polymorphisms (*APOA1* gene) and the development of dyslipidemia in schizophrenia patients on antipsychotic medications. The method is appropriate for investigating genetic associations with specific health outcomes, as gene polymorphisms are best studied through genetic association studies.  3. Were the cases recruited in an acceptable way?  Answer: Yes participants recruited according to well-defined inclusion/exclusion criteria.  4. Were the controls selected in an acceptable way?  Answer: Yes, the controls were matched for key characteristics such as age, gender, and other relevant factors (e.g., comorbidities, lifestyle).  5. Was the exposure accurately measured to minimize bias?  Answer: Yes. The exposure—antipsychotic drug treatment—was clearly defined, and the dyslipidemia diagnosis was based on standard clinical measures .  6. (a) Aside from the exposure, did the groups have similar characteristics?  Answer: Yes, the cases and controls had similar baseline characteristics such as age, gender, smoking status, physical activity.  (b) Have the authors taken account of the potential confounding factors in the design and/or in their analysis?  Answer: Yes, logistic regression analyses was used.  7. Was the treatment effect large?  Answer: Yes, the "treatment effect" I was large, there was a statistically significant and substantial difference in triglyceridaemia risk between individuals with rs5072 variant compared to those without. Odds ratio 1.50, 95%  confidence interval [CI]: 1.03, 2.17; P<0.05  8. Was the estimate of the treatment effect precise?  Answer: Yes, p <0.05.  9. Do you believe the results?  Answer: Yes, they are statistically robust, with the main finding of triglyceridaemia and rs5072 variant having a p<0.05, with regression analyses to account for potential confounding factors.  10. Can the results be applied to your patients/the population of interest?  Answer: Not fully, the population are Chinese ethnicity.  11. Do the results of this study fit with other available evidence?  Answer: Yes there is existing research that aligns with these findings. |
| SUMMARY  Positive/Methodologically Sound Aspects:  Clear Focus: The study investigates a specific, relevant question about genetic factors in antipsychotic-induced dyslipidemia.  Case-Control Design: This is an appropriate method for exploring genetic associations with health outcomes.  Exposure and Outcome Measurement: The measurement of antipsychotic exposure and lipid abnormalities (dyslipidemia) accurate and standardised, minimizing bias.  Negative/Relatively Poor Methodology:-  Unknown Aspects:  Confounder Control: While the design controls for some confounders, it’s unclear whether all relevant variables were accounted for (e.g., diet, exercise, comorbidities).  External Validity: The results would need to be validated in different populations to assess whether they can be generalized beyond the study sample. |

### **JBI Critical appraisal checklist for Case Report**

| Improvements in body composition, anthropometric measurements, and lipid profile following discontinuation of clozapine (Wysokinski (2015) |
| --- |
| 1. Were the patient’s demographic characteristics clearly described?  Answer: Yes. The demographic characteristics of the patient were described- age, sex, ethnicity were clearly defined.  2. Was the patient’s history clearly described and presented as a timeline?  Answer: Yes, the history was presented on a chronological timeline, starting from the diagnosis of schizophrenia,followed by the initiation of clozapine, the duration of use, and any notable changes in body composition, lipid levels, and other relevant outcomes during the treatment period, and once clozapine was discontinued.  3. Was the current clinical condition of the patient on presentation clearly described?  Answer: Yes. The clinical condition at the time of presentation was described including the patient’s weight, lipid profile, waist circumference, body mass index (BMI).  4. Were diagnostic tests or assessment methods and the results clearly described?  Answer: Yes. The study gave clear details on the meathods used to assess body composition ( weight, BMI, waist-circumference), lipid profiles (e.g., cholesterol, LDL, HDL levels, triglycerides).The results of these tests were presented with baseline values and post-discontinuation follow-up values to show the effects of clozapine cessation.  5. Was the intervention(s) or treatment procedure(s) clearly described?  Answer: Yes. The discontinuation of clozapine was clearly described.  6. Was the post-intervention clinical condition clearly described?  Answer: Yes. The post-intervention (post-discontinuation) clinical condition should was clearly described, focusing on the changes observed in body composition, lipid profile, and other metabolic parameters, with numerical data comparing pre- and post-intervention outcomes.  7. Were adverse events (harms) or unanticipated events identified and described?  Answer: Yes. A week after discontinuation of clozapine, the patient experienced insomnia, dysphoria and hypomania.  8. Does the case report provide takeaway lessons?  Answer: Yes. The case report should provide valuable lessons regarding the potential metabolic benefits of clozapine discontinuation, particularly for patients who experience weight gain and lipid abnormalities as a result of the drug.  It also highlights the importance of monitoring metabolic parameters in patients on clozapine, the potential improvements following discontinuation, and the importance of individualised treatment planning.  OVERALL APPRAISAL : INCLUDE |

### Critical appraisal tool for prognostic studies, based on CEBM guideline

| Long-term safety and effectiveness of lurasidone in schizophrenia: a 22-month, open-label extension study, Correl *et al.*, (2016) |
| --- |
| 1. Was the defined representative sample of patients assembled at a common (usually early) point in the course of their disease?  The study included patients from an earlier randomized controlled trial (RCT), which means that the sample was not recruited from the start of their disease but rather from those who had already participated in the prior trial. Therefore, it’s important to consider that these participants may not be at a "common" or early stage of their schizophrenia, as they were already being treated with lurasidone.  2. Was patient follow-up sufficiently long and complete?  Follow-up was for 22 months, which is quite long for a study investigating long-term outcomes such as safety and effectiveness.  3. Were outcome criteria either objective or applied in a ‘blind’ fashion?  Outcome criteria in the study were objective- mainly clinical measures (e.g., symptom reduction, safety data, and metabolic effects). While objective measures such as symptom scales (e.g., PANSS for schizophrenia symptoms) and metabolic parameters (e.g., weight, blood glucose, cholesterol levels) were used, the study was open-label. So, both participants and investigators were aware of the treatment being administered, and therefore blinding was not used.  Overall, while the outcome criteria were based on standard objective clinical measures, the lack of blinding could introduce some bias into how outcomes were assessed and reported, especially in subjective measures (e.g., self-reported symptoms or side effects).  4.. If subgroups with different prognoses are identified, did adjustment for important prognostic factors take place?  The study did not involve randomization in the open-label extension phase, meaning participants were not assigned to different subgroups with varying treatments.  The paper does mention the consideration of baseline factors (e.g., symptom severity and prior treatment history) and how they might influence outcomes, but it does not explicitly state whether statistical adjustments were made for these factors in the analysis. |

### **Initial codes generated during thematic analysis.**

| Paper Title (First Author, year) |  |  |  |  |  |
| --- | --- | --- | --- | --- | --- |
| Aripiprazole and Risperidone Present Comparable Long-Term Metabolic Profiles: Data From a Pragmatic Randomized Controlled Trial in Drug-Naïve First-Episode Psychosis  (Vázquez-Bourgon, 2022) | T 4 and Waist:Hip Ratio as Biomarkers of Antipsychotic-Induced Weight Gain in Han Chinese Inpatients with Schizophrenia (Li, 2018) | Improvements in Body Composition, Anthropometric Measurements, and Lipid Profile Following Discontinuation of Clozapine (Wysokinski 2015) | Pharmacogenetic Study of Antipsychotic-Induced Lipid and BMI Changes in Chinese Schizophrenia Patients: A Genome-Wide Association Study (Wong, 2024) | **Metabolic Adverse Effects of Antipsychotic Drugs in Patients with Schizophrenia (Althanoon, 2021)** | **Metabolic Disturbances Independent of Body Mass in Patients with Schizophrenia Taking Atypical Antipsychotics (Kang, 2015)** |
| Aripiprazole  Risperidone  Lipid profile  Long-term outcomes  Metabolic effect  Anthropometric  Dyslipidaemia | Biomarkers  Anthropometric Metabolic effect  Lipid profile  Olanzapine  Risperidone  Hormone | Clozapine  Lipid profile  Metabolic effect  Anthropometric  Complication | Pharmacogenetics  Clozapine  Olanzapine  Aripiprazole  Risperidone  Amisulpride  Quetiapine  Paliperidone | Metabolic effect  Olanzapine  Aripiprazole  Hormone  Lipid profile | Metabolic effect  Clozapine  Olanzapine  Quetiapine  Risperidone  Aripiprazole  Ziprasidone |
| Paper Title (First Author, year) |  |  |  |  |  |
| Effects of Atypical Antipsychotics on Serum Asprosin Level and Other Metabolic Parameters in Patients with Schizophrenia (Amini, 2024) | Circulating IGFBP-2 Levels Reveal Atherogenic Metabolic Risk in Schizophrenic Patients Using Atypical Antipsychotics (Nolin, 2020) | Long-Term Safety and Effectiveness of Lurasidone in Schizophrenia: A 22-Month, Open-Label Extension Study (Correl et al., 2016) | 1High Circulating MIF Levels Indicate the Association with Atypical Antipsychotic-Induced Adverse Metabolic Effects (Chen, 2024) | Association Between *APOA1* Gene Polymorphisms and Antipsychotic Drug-Induced Dyslipidemia in Schizophrenia (Fan, 2021) | Association of Blood Cell Counts with the Risk of Olanzapine- or Clozapine-Induced Dyslipidemia in Chinese Schizophrenia Patients (Xiong, 2019) |
| Asprosin  Metabolic effect  Biomarker  Olanzapine  Risperidone  Aripiprazole | IGFBP-2  Biomarker  Atherogenic  Metabolic  Olanzapine  Risperidone  Complication | Lurasidone  Long term outcomes  Metabolic effect | macrophage migration inhibitory factor (MIF)  Metabolic effect  Biomarker  Olanzapine  Clozapine  Aripiprazole  Risperidone  Quetiapine | *APOA1* Gene Polymorphisms  Dyslipidaemia  Pharmacogenetics | Blood cell count  Biomarker  Dyslipdeamia  Clozapine  Olanzapine |

### **Initial codes and their descriptions, generated during thematic analysis for each included study.**

| Lipid profile | A blood test that measures the levels of specific lipids (fats) in the blood, including cholesterol (HDL, LDL) and triglycerides |
| --- | --- |
| Long-term outcomes | The long-term effects or consequences that result from a specific condition, treatment, or intervention |
| Metabolic effect | Glucose and lipid levels |
| Dyslipidaemia | Abnormal levels of lipids in the blood, including high cholesterol, high triglycerides, or low levels of good cholesterol (HDL) |
| Biomarkers | Measurable substances in the body that indicate the presence or progression of a disease or the effect of a treatment |
| Anthropometric | Involves the measurement of the human body, typically to assess body composition and health. Common measurements include height, weight, waist circumference, and skinfold thickness. |
| Hormone | Chemical messengers in the body that regulate a wide range of physiological processes, including metabolism |
| Atherogenic | The process of developing atherosclerosis, which is the buildup of fatty deposits (plaque) in the arteries |
| Asprosin | A recently discovered hormone involved in regulating glucose and lipid metabolism |
| IGFBP-2 | Insulin-like Growth Factor Binding Protein 2 (IGFBP-2) is a protein that binds insulin-like growth factors (IGFs) and regulates their activity. |
| MIF  **(Macrophage Migration Inhibitory Factor)** | MIF is a cytokine involved in immune system regulation and inflammation |
| Pharmacogenetics | The study of how genetic variations affect individual responses to drugs. |
| *APOA1* Gene | The *APOA1* gene encodes the protein apolipoprotein A1, which is a major component of high-density lipoprotein (HDL) cholesterol |
| Blood cell count | The measurement of the number of different types of blood cells in a sample of blood, typically including red blood cells (RBCs), white blood cells (WBCs), and platelets. |
| Complication | Consequence of AA-induced dyslipidaemia |

### Themes generated from codes during thematic analysis.

| Major Themes | Relevant codes |
| --- | --- |
| 1. Association of atypical antipsychotics with dyslipidaemia/metabolic effect | Lipid profile  Dyslipidaemia  Metabolic effect |
| 2. Consequences of AA-induced dyslipidaemia | Long-term outcomes  Atherogenic  Complication |
| 3.Potential Biomarkers | Hormone  Anthropometry  Asprosin  IGFBP-2  MIF  Blood cell counts |
| 4. Pharmacogenetics | Pharmacogenetics  *APOA1* gene |
| Minor Themes | Relevant codes |
| Distribution of research | Clozapine  Olanzapine  Quetiapine  Risperidone  Aripiprazole  Ziprasidone  Lurasidone  Paliperidone |
| Anthropometry | Waist-to-hip ratio  Body weight |
